# A minimum-labeling approach for reconstructing protein networks across multiple conditions

**DOI:** 10.1186/1748-7188-9-1

**Published:** 2014-02-09

**Authors:** Arnon Mazza, Irit Gat-Viks, Hesso Farhan, Roded Sharan

**Affiliations:** 1 Blavatnik School of Computer Science, Tel Aviv University, 69978 Tel Aviv, Israel; 2 Department of Cell Research and Immunology, Tel Aviv University, 69978 Tel Aviv, Israel; 3 Biotechnology Institute Thurgau, University of Konstanz, Unterseestrasse 47, CH-8280 Kreuzlingen, Switzerland

**Keywords:** Protein-protein interaction networks, Graph algorithms, Integer linear programming

## Abstract

**Background:**

The sheer amounts of biological data that are generated in recent years have driven the development of network analysis tools to facilitate the interpretation and representation of these data. A fundamental challenge in this domain is the reconstruction of a protein-protein subnetwork that underlies a process of interest from a genome-wide screen of associated genes. Despite intense work in this area, current algorithmic approaches are largely limited to analyzing a single screen and are, thus, unable to account for information on condition-specific genes, or reveal the dynamics (over time or condition) of the process in question.

**Results:**

We propose a novel formulation for the problem of network reconstruction from multiple-condition data and devise an efficient integer program solution for it. We apply our algorithm to analyze the response to influenza infection and ER export regulation in humans. By comparing to an extant, single-condition tool we demonstrate the power of our new approach in integrating data from multiple conditions in a compact and coherent manner, capturing the dynamics of the underlying processes.

## Background

With the increasing availability of high-throughput data, network biology has become the method of choice for filtering, interpreting and representing these data. A fundamental problem in network biology is the reconstruction of a subnetwork that underlies a process of interest by efficiently connecting a set of implicated proteins (e.g. derived by some genome-wide screen) in a network of physical interactions. In recent years, several algorithms have been suggested for different variants of this problem, including the Steiner tree based methods of [1,2], the flow based approach of [3] and the anchored reconstruction method of [4].

Despite the plethora of network reconstruction methods, these have been so far largely limited to explaining a single experiment or condition. In practice, the network dynamically changes over time or conditions, calling for reconstructions that can integrate such data to a coherent picture of the activity dynamics of the underlying pathways.

Here we tackle this multiple-condition scenario, where the reconstructed subnetwork should explain in a coherent manner multiple experiments driven by the same set of proteins (referred to here as *anchor* proteins) while producing different sets of affected proteins, or *terminals*. As in the single-condition case, a parsimonious assumption implies that the reconstructed subnetwork should be of minimum size. In addition, we require that its pathways, leading from the anchors to each of the terminals, are as homogeneous as possible in terms of the conditions, or *labels* they span. We formulate the resulting minimum labeling problem, show that it is NP complete and characterize its solutions. We then offer an equivalent formulation that allows us to design a polynomial integer linear programming (ILP) formulation for its solution. We implement the ILP algorithm, *MKL*, and apply it to two datasets in humans concerning the response to influenza infection and ER export regulation. We show that the MKL networks are significantly enriched with respect to the related biological processes and allow obtaining of novel insights on the modeled processes. Finally, we compare MKL with an extant method, ANAT [4], demonstrating the power of our algorithm in integrating data from multiple conditions in a compact and informative manner.

## Preliminaries

Let *G* = (*V*, *E*) be a directed graph, representing a protein-protein interaction (PPI) network, with vertex set *V* and edge set *E*, and let *a* ∈ *V* be an anchor node. Denote by *In*(*v*) (*Out*(*v*)) the set of incoming (outgoing) edges of a node *v* ∈ *V*, respectively. Let *L* = {1, …, *k*} be a set of labels, representing *k* ≥ 1 conditions. Let *f*:*E* → 2^(*L*)^ be a labeling function that assigns each edge of *E* a (possibly empty) subset of labels. For 1 ≤ *i* ≤ *k*, we define *E*_*i*_(*f*) := {*e* ∈ *E* : *i* ∈ *f*(*e*)} to be the set of edges with label *i*. We further denote $f_{\text{in}}(v)=\underset{e\in \text{In}(v)}{⋃}f(e)$ and $f_{\text{out}}(v)=\underset{e\in \text{Out}(v)}{⋃}f(e)$.

We say that a labeling function *f* is *valid* if for every terminal *t* and condition *i* in which *t* is affected, there exists a path from *a* to *t* whose edges are restricted to *E*_*i*_(*f*), or in other words, are assigned with the label *i*. We evaluate the *cost* of the labeling according to the number of labels *L*(*f*) used and the number of edges *N*(*f*) that are assigned with at least one label. Formally, $L(f)=\underset{e\in E}{\sum }|f(e)|$ and *N*(*f*) = |{*e* ∈ *E* : *f*(*e*) ≠ ∅}|. The cost is then defined as *α* · *L*(*f*) + (1 - *α*) · *N*(*f*), where 0 ≤ *α* ≤ 1 balances the two terms.

We study the following **minimum***k-***labeling (MKL)** problem on *G*: The input is an anchor node *a* ∈ *V* and *k* ≥ 1 sets of terminals *T*_1_, …, *T*_*k*_ in *V* ∖ {*a*} that implicitly assign to each terminal the subset of conditions (or labels) in which it is affected. The objective is to find a valid labeling of the edges of *G* of minimum cost.

**Figure 1:**
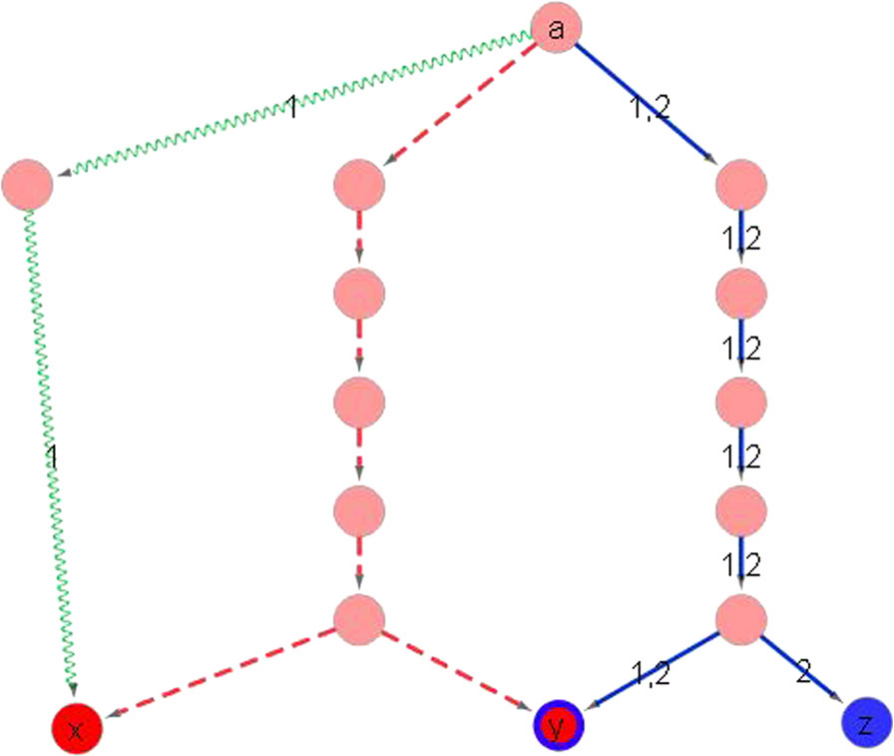
**The optimal MKL solution for *α *= 0.5 is neither the union of label-specific Steiner trees nor a subgraph of it.** In this instance *k* = 2, *T*_1_ = {*x*, *y*} and *T*_2_ = {*y*, *z*}. The optimal Steiner trees for *T*_1_ and *T*_2_ are composed of the red (dashed) and blue (solid) edges, respectively. The best MKL solution that uses only edges of the union can be achieved by pushing label 1 over the red edges and 2 over the blue edges, resulting in 14 labels and 14 edges. In contrast, the optimal solution, whose labels appear on top of the figure, contains the blue and green (waved) edges, spanning 15 labels and 9 edges.

**Figure 2:**
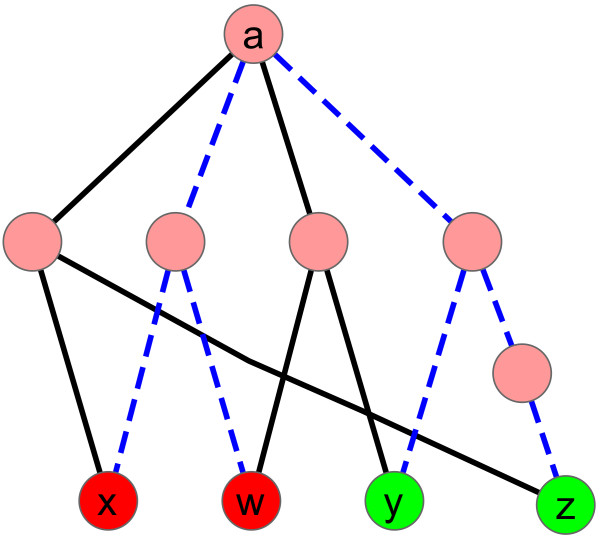
**The optimal MKL solution for *α *= 0.6 is not a minimum Steiner tree over all terminals.** In this instance *k* = 2, *T*_1_ = {*x*, *w*} and *T*_2_ = {*y*, *z*}. The black (solid) edges form a Steiner tree with 6 edges and 8 labels, whereas the blue (dashed) edges constitute an MKL solution with 7 edges and 7 labels.

Clearly, any valid labeling induces a subnetwork that can model the given conditions: this subnetwork is comprised of those edges that are assigned a non-empty subset of labels. We note that for *k* = 1 we have *L*(*f*) = *N*(*f*), thus in this case the MKL problem is equivalent to the minimum directed Steiner tree problem. The parameter *α* balances between two types of solutions: (1) a subnetwork with minimum number of labels (*α* = 1), which is equivalent to the union of independent Steiner trees for each of the conditions, and (2) a subnetwork with minimum number of edges (*α* = 0), which is simply a Steiner tree spanning the terminals in the union of all conditions. However, general instances of MKL where *α* ≠ 0, 1 can be solved neither by combining the independent Steiner trees of each of the conditions nor by constructing a single Steiner tree over all terminals. This is illustrated by the toy examples in Figures 1 and 2. Next, we provide a characterization of solutions to the MKL problem.

### 

**Theorem 1.** Given a solution labeling *f* to an MKL instance, let *G*_*i*_ denote the subgraph of *G* that is induced by the edges in *E*_*i*_(*f*). Then *G*_*i*_ is a directed tree rooted at *a*.

### 

*Proof*. By definition, there is a directed path in *G*_*i*_ from *a* to each of the terminals in *T*_*i*_. Clearly, any edge directed into *a* can be removed without affecting the constraints of a valid solution. Thus, it suffices to show that the underlying undirected graph of *G*_*i*_ contains no cycles. By minimality of the solution, every vertex in *G*_*i*_ is reachable from *a* or else it can be removed along with its edges. Suppose to the contrary that *v*_1_, …, *v*_*n*_ is a cycle in the underlying graph. Since *a* cannot be on this cycle and by the above observation, each of the cycle’s vertices is reachable from *a*. W.l.o.g., let *v*_1_ be the farthest from *a* in *G*_*i*_ among all cycle vertices. Then one can obtain a smaller solution by removing one of the edges (*v*_1_, *v*_2_), (*v*_*n*_, *v*_1_) (depending on their orientations), a contradiction.

As noted earlier, when *k* = 1 the MKL problem is equivalent to the minimum directed Steiner tree problem, which is known to be NP-complete [5]. A simple reduction from this case yields the following result:

### 

**Theorem 2.** The MKL problem is NP-complete for every *k* ≥ 1.

### 

*Proof*. Let *k* > 1. Given an instance of the minimum 1-labeling problem, that is, a network *G* = (*V*, *E*), an anchor *a* ∈ *V* and a single set of terminals *T* ⊂ *V*, we generate the following input to the minimum *k*-labeling problem. Define the background network *G*^′^ = (*V*^′^, *E*^′^), where *V*^′^ = *V* ∪ {*t*_1_, …, *t*_*k*-1_} and *E*^′^ = *E* ∪ {(*a*, *t*_1_),…,(*a*,*t*_*k*-1_)}, where ${\left\{t_{i}\right\}}_{1}^{k-1}$ are new nodes not in *V*. The input *k* sets of terminals are then *T*, {*t*_1_}, …, {*t*_*k*-1_}, and the anchor remains *a*. The key observation to complete this proof is that an optimum solution to the reduced instance must include all edges (*a*, *t*_*i*_), plus an optimal tree that connects *a* to the terminals in *T* using a single label.

## Methods

### An alternative formulation of MKL

As the MKL problem is NP-complete, we aim to design an integer linear program for it, which will allow us to solve it to optimality or near-optimality for moderately-sized instances. In order to design an efficient ILP, we first provide an alternative formulation of the MKL problem, expressed in terms of units of flow per label pushed from the anchor toward the terminals. To this end, we extend the labeling definition to support assignment of multi-sets, as described below. We denote a multi-set by a pair *M* = 〈 *S*, *μ* 〉, where *S* is a set and $\mu :S\to Z^{+}$. We say that *x* ∈ *M* if *x* ∈ *S*. We let |*M*| denote the cardinality of the underlying set *S*.

The union ⊎ of two multi-sets 〈*S*_1_, *μ*_1_〉, 〈*S*_2_, *μ*_2_〉 is defined as the pair 〈*S*, *μ*〉, where *S* = *S*_1_∪*S*_2_; for every *x* ∈ *S*_1_ ∩ *S*_2_, *μ*(*x*) = *μ*_1_(*x*) + *μ*_2_(*x*); for *x* ∈ *S*_1_ ∖ *S*_2_, *μ*(*x*) = *μ*_1_(*x*); and for *x* ∈ *S*_2_ ∖ *S*_1_, *μ*(*x*) = *μ*_2_(*x*). We extend the definitions of *f*_*in*_(*v*) and *f*_*out*_(*v*) to multi-sets using this union operator. Finally, for a vertex *v* ≠ *a* we let *L*(*v*) = {*i* ∈ *L*:*v* ∈ *T*_*i*_}; note that for non-terminal nodes *L*(*v*) = ∅.

The alternative objective formulation is as follows: Find a multi-set label assignment *g*:*E* → 2^(*L*)^ that satisfies the following constraints: 

(i) *g*_*out*_(*a*) = 〈*L*, *μ*〉, where *μ*(*i*) = |*T*_*i*_| for every *i* ∈ *L*.

(The total amount of flow that goes out from the anchor per label equals the number of terminals that belong to the corresponding experiment).

(ii) For every *v* ≠ *a*, *g*_*in*_(*v*) = *g*_*out*_(*v*) ⊎ *L*(*v*). (For each label *i*, the incoming flow of a node *v* equals its outgoing flow, incremented by 1 if *v* is a terminal expressed in experiment *i*).

(iii) Denote $L(g)=\underset{e\in E}{\sum }|g(e)|$, *N*(*g*) = |{*e* ∈ *E* : *g*(*e*) ≠ ∅}|, and let 0 ≤ *α* ≤ 1. Then *α* · *L*(*g*) + (1 - *α*) · *N*(*g*) is minimal.

We claim that the two formulations are equivalent. Given a multi-set labeling *g*, it is easy to transform it into a labeling *f* by taking at each edge the underlying set of labels. One can show that the labeling *f* is valid, i.e. for each *i* there are paths in *E*_*i*_(*f*) that connect *a* to each of the terminals in *T*_*i*_. For the other direction, given a labeling *f* we can transform it into a multi-set labeling *g* by defining the multiplicity of a label *i* at the edge (*u*, *v*) ∈ *E*_*i*_(*f*) as the number of terminals from *T*_*i*_ in the subtree of *G*_*i*_ that is rooted at *v*. It is easy to see that all constraints are satisfied by this transformation.

The above problem formulation can be made stricter by requiring that the set of incoming labels to a terminal is exactly the set of labels associated with the terminal. That is, for every terminal *t* and *i* ∈ *L* ∖ *L*(*t*), we require that *i* ∉ *g*_*in*_(*t*). Our ILP formulation includes this requirement in order to better reflect the experimental observations, though in practice both versions produce very similar results.

### An ILP algorithm

In order to formulate the problem as an integer program, we define three sets of variables: (i) binary variables of the form $y_{e}^{i}$, indicating for every *e* ∈ *E* and *i* ∈ *L* whether the edge *e* is tagged with label *i*; (ii) integer variables of the form $x_{e}^{i}$, indicating for every *e* ∈ *E* and *i* ∈ *L* the multiplicity of label *i* (in the range 0 to |*T*_*i*_|); and (iii) binary variables of the form *z*_*e*_, indicating for every *e* ∈ *E* whether the edge *e* participates in the subnetwork (carrying any label). For a vertex *v* ∈ *V*, let $b_{v}^{i}$ be a binary indicator of whether *i* ∈ *L*(*v*) or not. Let *α* be some fixed value in the range [0,1]. The formulation is as follows (omitting the constraints on variable ranges):

(1) $$\begin{array}{ll} \min\alpha \cdot \underset{e\in E,i\in L}{\sum }\overset{i}{\underset{e}{y}}+(1-\alpha )\cdot & \underset{e\in E}{\sum }z_{e}\\ \text{s.t.:} & \\ y_{e}^{i}\leq x_{e}^{i}\leq |T_{i}|\cdot y_{e}^{i} & \forall e\in E,i\in L \end{array}$$

(2) $$\begin{array}{ll} y_{e}^{i}\leq z_{e} & \;\forall e\in E,i\in L \end{array}$$

(3) $$\begin{array}{ll} \underset{e\in \text{Out}(a)}{\sum }x_{e}^{i}=|T_{i}| & \;\forall i\in L \end{array}$$

(4) $$\begin{array}{ll} \underset{e\in \text{In}(v)}{\sum }x_{e}^{i}=\underset{e\in \text{Out}(v)}{\sum }x_{e}^{i}+b_{v}^{i} & \forall v\in V\setminus \{a\},i\in L \end{array}$$

(5) $$\begin{array}{ll} \underset{e\in \text{In}(t)}{\sum }y_{e}^{i}=0 & \;\forall t\in T,i\notin L(t) \end{array}$$

By Theorem 1, the constraint

(6) $$\underset{e\in \text{In}(v)}{\sum }y_{e}^{i}\leq 1\;\;\forall v\in V,\;i\in L$$

can be added to the ILP without affecting the optimal solution. The following Lemma leverages this insight for enhancing the ILP performance by removing some of the integrality constraints.

#### 

**Lemma 1.** Assume that constraint (6) is added to the ILP formulation above. If all $y_{e}^{i}$’s are restricted to binary values then the range constraints $x_{e}^{i}\in [0,|T_{i}|]$ and *z*_*e*_∈[0,1] guarantee that all $x_{e}^{i}$’s and *z*_*e*_’s are assigned integer values in any optimal solution.

#### 

*Proof*. Let *v* ∈ *V*. We first prove that for every *e* ∈ *In*(*v*) and *i* ∈ *L*, $x_{e}^{i}$ must be an integer. By the new constraint (6) and the integrality of all $y_{e}^{i}$’s, the sum $\underset{e\in \text{In}(v)}{\sum }y_{e}^{i}$ is either 0 or 1. If it is 0 then by constraint (1), for each of these edges $x_{e}^{i}=0$. Otherwise, exactly one of these edges has $y_{e}^{i}=1$ and therefore $x_{e}^{i}>0$. Denote by *G*_*i*_ the subnetwork that is induced by all edges having nonzero flow for label *i* (i.e. edges *e* fulfilling $x_{e}^{i}>0$). Denote by *T*_*i*_(*v*) the set of terminals in *T*_*i*_ that are reachable from *v* in *G*_*i*_, and let *t* ∈ *T*_*i*_(*v*). By applying the above argument for each of the nodes between *v* and *t*, we infer that there is a single path that carries flow from *v* to *t* in *G*_*i*_, and that all of *t*’s incoming flow (of label *i*) must pass through *v*. Every *t* ∈ *T*_*i*_(*v*) absorbs a flow of 1 and therefore from the flow-preserving constraint (4), $\underset{e\in \text{In}(v)}{\sum }x_{e}^{i}\geq |T_{i}(v)|$. The other direction holds too since the flow of label *i* that *v* sends can be collected only by terminals in *T*_*i*_(*v*). Thus, we conclude that all $x_{e}^{i}$’s in this sum equal 0 except for a single element which equals |*T*_*i*_(*v*)|, i.e. all of them are integers.

To prove that all *z*_*e*_’s are integral, consider some edge *e* ∈ *E*. If there exists *i* ∈ *L* such that $y_{e}^{i}=1$ then from constraint (2) it follows that *z*_*e*_ = 1. Otherwise, the equality *z*_*e*_ = 0 follows from the minimality of the solution.

### Heuristic data reduction and runtime analysis

Since solving an ILP is a time consuming task, we devised a heuristic method for filtering the input network, aiming to capture those edges that the MKL optimal solution is more likely to use. Specifically, we focused on (directed) edges that lie on a near shortest path – up to *d* edges longer than a shortest path – between the anchor and any of the terminals.

**Figure 3:**
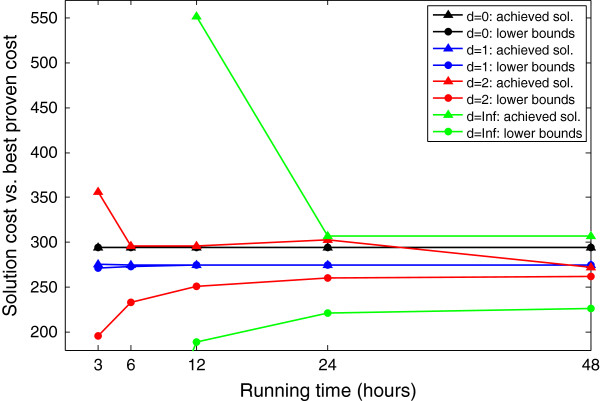
**Dependency of the MKL algorithm performance on the heuristic filtering.** This figure compares the performance of the MKL algorithm with respect to different values of the heuristic data reduction parameter *d* and when setting different limits on the running time. For each value of *d* (0, 1, 2 or no heuristic filtering), two plots with the same color are displayed: the top plot (triangle symbols) shows the cost of the achieved solution after the specified number of hours (or less); the bottom plot (circles) shows the best proven theoretical lower bound on an optimal solution as reported by the same execution. Note that for *d* = 0 these two (black) plots fully coincide.

In order to support this heuristic and find a value for *d* that achieves a satisfying balance between running time and optimality, we tested the performance of our ILP algorithm on the influenza dataset (which is the more computationally expensive dataset described in the Experimental results Section) with *d* = 0, *d* = 1, *d* = 2, and without the heuristic filtering. These parameter values induced input background networks of 0.01*x*, 0.1*x*, 0.5*x* and *x* edges, respectively, where *x*∼80,000 is the complete network size. Using *d* = 1, six hours were sufficient to achieve an optimal solution of cost (combined number of labels and edges) 275. Using *d* = 2, a solution of similar quality (cost 272) was achieved after 48 hours. This execution also proved that the optimal solution with *d* = 2 has a lower bound of at least 262, showing that the theoretical improvement over *d* = 1 is limited to less than 5%. This analysis motivated our selection of *d* = 1 for the experimental evaluation that follows. Further, it is interesting to note that with this choice, the convergence toward the optimum is very fast: in three hours one could achieve a solution that is less than 1% behind the optimum (though this time period was not enough to prove this approximation guarantee). This is in large contrast to the settings of *d* ≥ 2 that are characterized by very slow convergence (> 10% approximation ratio after 24 hours). The results are summarized in Figure 3.

### Performance evaluation

We used the commercial IBM ILOG CPLEX optimizer to solve the ILP and instructed it to accept approximate solutions that deviate by at most 5% from the optimum, enabling our executions to end within less than two hours.

We evaluated a solution subnetwork using both network-based and biological measures. The network-based measures included the number of labels, number of edges and a *homogeneity* score. To compute the homogeneity score of a node *v*, we examined the frequencies of all subsets of labels assigned to terminals under *v*. The score of *v* was defined as the highest frequency found divided by the number of terminals under *v*. The homogeneity score of the subnetwork was then defined as the average over all nodes that span at least two terminals. To quantify the biological significance of the reconstructed subnetworks, we measured the functional enrichment of their internal nodes (non-input nodes) with respect to validation sets that pertain to the process in question. In addition, we provide expert analysis of the subnetworks.

We compared the performance of our method to that of the state-of-the-art ANAT reconstruction tool [4], which was shown to outperform many existing tools in anchored reconstruction scenarios. For each dataset, we applied ANAT (with its default parameters, and without the heuristic filtering) to each condition separately, then unified the results to get an integrated subnetwork. We labeled the solution straightforwardly: an edge *e* was labeled *i* if *e* participated in the subnetwork that was constructed for condition *i*. We also compared our results to those attained by computing a Steiner tree over the terminals of all conditions together, implemented using the same ILP algorithm by setting *α* = 0.

## Experimental results

We tested the performance of our algorithm on two human datasets concerning the cellular response to the influenza virus and ER export regulation. The two datasets were analyzed in the context of a human PPI network reported in [4] which contains 44,738 (bidirectional) interactions over 10,169 proteins.

For each of the two datasets, we tested the robustness of our algorithm to different choices of the weighting parameter *α*, observing that the number of edges and labels varied by at most 8% and 4%, respectively, over a wide range of values (0.25 – 0.75). Thus, we chose *α* = 0.5 for our analyses in the sequel.

### Response to influenza infection

We used data on the response to viral infection by the H1N1 influenza strain A/PR/8/34 (‘PR8’) in primary human bronchial epithelial cells [6]. The dataset contains a collection of 135 virus-human PPIs and gene expression profiles, measured at different time points along the course of the infection. We focused on four time points (the “conditions”) *t* = 2, 4, 6, 8 (i.e. *k* = 4 labels), in each time point selecting those genes that were differentially expressed above a cutoff of 0.67 [6]. We did not include time points earlier than *t*=2 or later than *t* = 8, as the former had no or very few differentially expressed genes, while the latter induced an order of magnitude larger gene sets that are presumably associated with secondary responses.

We augmented the human network by the influenza-host PPIs and an auxiliary anchor node (named ‘virus’) which we connected to the 10 viral proteins. After the heuristic filtering (using *d* = 1), the network contained 1,598 proteins and 8,708 interactions.

**Figure 4:**
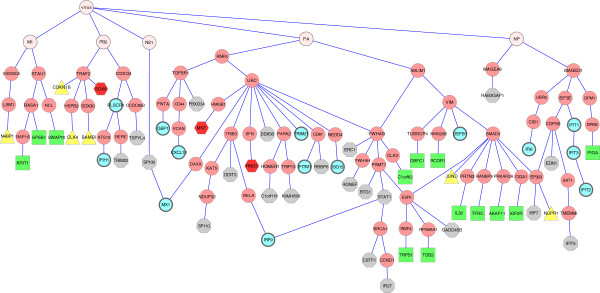
**The MKL subnetwork for the influenza infection data.** Terminal nodes are marked by their corresponding time point: t = 2 - yellow/triangle; t = 4 - green/square; t = 6 - red/hexagon; t = 8 - gray/octagon; more than one time point - cyan oval nodes with thick border. The root is the artificial virus node and the first level is composed solely of viral proteins.

The four terminal sets contained 8,19,19 and 49 proteins, respectively, with 77 total in their union, out of which 57 were reachable from the anchor. The resulting MKL subnetwork, which is shown in Figure 4, contains 127 edges over 123 nodes (117 human, 5 viral and the anchor node) with 60 internal (non-input) nodes. This subnetwork is much more compact than the solution suggested by ANAT, which contains 173 nodes out of which 106 are internal. The subnetworks of MKL and ANAT are quite different in terms of node composition, having 31 internal intersecting nodes. A summary of our network-based measures for the subnetworks predicted by our algorithm, ANAT, and the Steiner tree algorithm is given in Table 1.

**Table 1:** Comparison of network-based measures between MKL, ANAT and the Steiner tree algorithm

**Measure**	**MKL**	**ANAT**	**Steiner**
***Influenza infection***			
No. of labels	158	254	187
No. of edges	122	186	113
Homogeneity score	0.63	0.58	0.57
***ER export***			
No. of labels	152	213	163
No. of edges	145	203	144
Homogeneity score	0.88	0.74	0.81

This table compares the subnetworks reconstructed by the MKL, ANAT and Steiner tree algorithms for the viral infecion and the ER export datasets with respect to the following measures: number of labels, number of edges and homogeneity score.

Next, we scored the enrichments of both subnetworks with viral infection related processes such as: viral reproduction, intracellular receptor mediated signaling pathway and apoptosis. The MKL subnetwork was highly enriched with these processes, outperforming the ANAT and the Steiner subnetworks (Table 2). In the following we present a detailed analysis of the MKL inferred subnetwork and demonstrate its high predictive power and its ability to characterize viral proteins and host mediators in terms of their temporal effect on their targets. Specifically, we show that this subnetwork suggests that an imbalance in the timing of effect between viral proteins (e.g. M1 and NP) or between host mediators (such as *Smad3* and *UBC*) can reveal their different kinetics of influence on host proteins. This is in large contrast to the results produced by the ANAT tool, which does not provide any timing imbalance among downstream targets of viral proteins or host mediators (data not shown).

**Table 2:** Comparison of enrichments between the MKL, ANAT and Steiner tree solutions

**Biological process**	**MKL**	**ANAT**	**Steiner**
***Influenza infection***			
Intracellular receptor mediated	6.5e-10	2.1e-04	1.2e-05
signaling pathway (GO:0030522)			
Apoptosis (GO:0006915)	3.7e-04	1.7e-04	3.3e-04
Viral reproduction (GO:0016032)	2.5e-03	>0.01	>0.01
***ER export***			
Vesicle-mediated transport	1.2e-05	7.6e-04	8.5e-05
(GO:0016192)			
Cellular membrane organization	1.4e-05	6.6e-05	1.6e-05
(GO:0016044)			
Intracellular protein transport	9.2e-06	7.8e-06	2.3e-05
(GO:0006886)			

This table compares the hypergeometric p-values indicating the significance of the overlap between each of the predicted subnetworks (considering non-input genes only) and the gene sets of GO categories that are of relevance to the investigated biological processes.

We first present an example of an inferred pathway, selected to demonstrate our MKL approach. The PA-*Rnf5*-*UBC*- *DAXX*-*MX1* and NS1-*SP100*-*MX1* paths are a clear example of a predicted pathway that is well supported by extant experimental findings. It is consistent with the known role of both *DAXX* and *SP100* as major components of the PML bodies which control together the localization of MX1 in distinct nuclear components [7]. Further, *DAXX* is known to be regulated *in vivo* by ubiquitination through *UBC* and *Rnf5*[8], supporting our placement of *DAXX* downstream to *UBC*.

The MKL network shows that the targets of some human proteins have a common temporal behavior, whereas others have different downstream temporal responses. This is consistent with the fact that PPIs naturally represent different mechanisms that might differ in their kinetics. For example, the targets of *Traf2* are mainly early responding genes whereas the targets of *Ccdc33* have longer temporal responses. The early effect of *Traf2* is consistent with the findings that *Traf2* is a signaling transduction kinase protein with fast kinetics. A similar characterization can be applied to other signal transduction proteins such as *Smad3*. Conversely, the *Ccdc33* protein regulates its targets in late time points (6–8 hours) by an unknown mechanism. The results here suggest that this mechanism is orders of magnitude slower than phosphorylation. Similarly, the control of *Rnf5* and *UBC* is expected to show fast kinetics through ubiquitination. Nevertheless, we find that all the *Rnf5* / *UBC* 19 targets are controlled in late time points (6–8 hours), suggesting a novel temporal (late) control on the activity of *Rnf5*-specific *UBC*-based ubiquitination during the course of influenza infection.

### Regulation of endoplasmic reticulum (ER) export

The journey of secretory proteins, which make up roughly 30% of the human proteome starts by exit from the ER. Export from the ER is executed by so called COPII vesicles that bud from ER exit sites (ERES). A protein that is of central importance for ERES biogenesis and maintenance is *Sec16A*, a large (~250 kDa) protein that localizes to ERES and interacts with COPII components [9]. We have recently performed a siRNA screen to test for kinases and phosphatases that regulate the functional organization of the early secretory pathway [10]. Among the hits identified were 64 kinases/phosphatases that when depleted result in a reduction in the number of ERES. Thus, these are 64 different potential regulators of ER export. More recently, a full genome screen tested for genes that regulate the arrival of a reporter protein from the ER to the cell surface [11]. There, the depletion of 45 proteins was shown to affect ERES. However, whether the defect in arrival of the reporter to the cell surface was due to an effect on ER export or due to alterations in other organelles along the secretory route (e.g., Golgi apparatus) remains to be determined.

**Figure 5:**
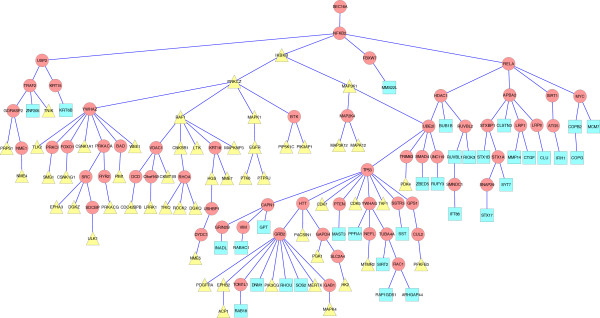
**The MKL subnetwork for the ER export data.** Terminal nodes are colored/shaped according to the screen they were discovered in: [10] - yellow/triangle, and [11] - cyan/square.

We applied MKL to these two screens, serving as two “conditions” highlighting different repertoires of ER export signaling-regulatory pathways. As the two screens do not intersect (most likely due to differences in read-outs), there were 109 terminals overall, 85 of them reachable in our human PPI network. Due to its central importance for ER export and ERES formation, we chose *Sec16A* as the anchor for this application. After the heuristic filtering, the network contained 1,907 nodes and 11,329 edges. The resulting MKL subnetwork, which has 145 nodes and 59 internal ones, is depicted in Figure 5. In comparison, the ANAT solution contains 190 nodes and 104 internal ones (with 35 internal nodes common to the two solutions). As evident from Table 1, the MKL solution has a substantially lower cost and is more homogeneous.

We assessed the functional enrichment of the MKL subnetwork with biological processes that are of relevance to ER export such as cellular membrane organization, intracellular protein transport and vesicle-mediated transport. All three categories were highly enriched, and the *p*-values attained compare favorably to those computed for the ANAT and the Steiner solutions (Table 2).

Interestingly, 4 proteins of the MKL solution are related to autophagy (two of them internal nodes, *p* = 0.02). Autophagy is an endomembrane-based cellular process that is responsible for capturing and degradation of surplus organelles and proteins. Links between ER export and autophagy have been proposed [12] but there is very limited mechanistic insight into this link. The vesicle-mediated transport process includes the *STX17*, *SNAP29* and *ULK1* proteins. The latter is a kinase that initiates the biogenesis of autophagosomes [13]. *STX17* and *SNAP29* were recently proposed to be involved in autophagy by promoting the formation of ER-mitochondria contact sites and the fusion of autophagosomes with lysosomes [14,15]. As the MKL network was generated with terminals and an anchor that regulate ER export, we propose that this approach could be used to identify the molecular link between secretion and autophagy in the future.

## Conclusions

The protein-protein interaction network represents a combination of diverse regulation and interaction mechanisms operating in different conditions and time scales. Integrating such data in a coherent manner to describe a process of interest is a fundamental challenge, which we aim to tackle in this work via a novel ILP-based minimum labeling algorithm. We apply our algorithm to two human datasets and show that it attains compact solutions that capture the dynamics of the data and align well with current knowledge. We expect this type of analysis to gain further momentum as composite datasets spanning multiple conditions and time points continue to accumulate.

## Competing interests

The authors declare that they have no competing interests.

## Authors’ contributions

AM and RS conceived the study and designed the algorithms. AM implemented the method and performed the computational experiments. IGV and HF performed the biological analyses. All authors read and approved the final manuscript.
